# Current Insights Into the Pathology of Canine Intervertebral Disc Extrusion-Induced Spinal Cord Injury

**DOI:** 10.3389/fvets.2020.595796

**Published:** 2020-10-27

**Authors:** Ingo Spitzbarth, Sarah A. Moore, Veronika M. Stein, Jonathan M. Levine, Bianca Kühl, Ingo Gerhauser, Wolfgang Baumgärtner, The Canine Spinal Cord Injury Consortium (CANSORT-SCI), Sarah A. Moore

**Affiliations:** Author Affiliations: DACVIM-Neurology, Associate Professor, Neurology and Neurosurgery, Department of Veterinary Clinical Sciences, The Ohio State University College of Veterinary Medicine, Columbus, OH, United States; DACVIM Neurology, Professor of Neurology/Neurosurgery; Distinguished Chair of Gerontology, Department of Clinical Sciences, North Carolina State University College of Veterinary Medicine, Raleigh, NC, United States; DACVIM-Neurology; Chair, and Head, Department of Small Animal Clinical Sciences, College of Veterinary Medicine and Biomedical Sciences, Texas A&M University, College Station, TX, United States; DAVCIM (Neurology), Assistant Professor of Neurology, Department of Veterinary Clinical Sciences, Purdue University College of Veterinary Medicine, West Lafayette, IN, United States; Professor Neurology & Neurosurgery; Professor in Small Animal Clinical Sciences, College of veterinary Medicine, Texas A&M University, College Station, TX, United States; Professor and Service Head, Neurology and Neurosurgery, Department of Veterinary Clinical Sciences, College of Veterinary Medicine, The Ohio State University, Columbus, OH, United States; Department of Clinical Sciences, Colorado State University, Fort Collins, CO, United States; Department of Clinical Science and Services, Royal Veterinary College, Hawkshead Lane, Hatfield, United Kingdom; The Royal Veterinary College, University of London, Hawkshead Lane, Hatfield, Hertfordshire, United Kingdom & CVS referrals, Bristol Veterinary Specialists at Highcroft, Bristol, United Kingdom; Faculty of Veterinary Medicine, Institute of Veterinary Pathology, Leipzig University, Leipzig, Germany; Division of Clinical Neurology, Department for Clinical Veterinary Medicine, Vetsuisse Faculty, University of Bern, Bern, Switzerland; Department Small Animal Medicine and Surgery, University of Veterinary Medicine Hannover, Hannover, Germany; Neurology and Neurosurgery, Department of Veterinary Medicine and Surgery, University of Missouri, University of Missouri, Columbia, MO, United States; Department of Small Animal Medicine and Surgery, University of Veterinary Medicine Hannover, Hannover, Germany; ^1^Faculty of Veterinary Medicine, Institute of Veterinary Pathology, Leipzig University, Leipzig, Germany; ^2^Department of Veterinary Clinical Sciences, The Ohio State University College of Veterinary Medicine, Columbus, OH, United States; ^3^Department for Clinical Veterinary Medicine, Vetsuisse Faculty, University of Bern, Bern, Switzerland; ^4^Department of Small Animal Clinical Sciences, College of Veterinary Medicine and Biomedical Sciences, Texas A&M University, College Station, TX, United States; ^5^Department of Pathology, University of Veterinary Medicine Hannover, Hanover, Germany

**Keywords:** spinal cord injury, IVDE, extrusion, macrophage, immunohistochemistry, axonal damage, macrophage polarization, cytokine

## Abstract

Spinal cord injury (SCI) in dogs is commonly attributed to intervertebral disc extrusion (IVDE). Over the last years substantial progress was made in the elucidation of factors contributing to the pathogenesis of this common canine disease. A detailed understanding of the underlying histopathological and molecular alterations in the lesioned spinal cord represents a prerequisite to translate knowledge on the time course of secondary injury processes into the clinical setting. This review summarizes the current state of knowledge of the underlying pathology of canine IVDE-related SCI. Pathological alterations in the spinal cord of dogs affected by IVDE-related SCI include early and persisting axonal damage and glial responses, dominated by phagocytic microglia/macrophages. These processes are paralleled by a pro-inflammatory microenvironment with dysregulation of cytokines and matrix metalloproteinases within the spinal cord. These data mirror findings from a clinical and therapeutic perspective and can be used to identify biomarkers that are able to more precisely predict the clinical outcome. The pathogenesis of progressive myelomalacia, a devastating complication of SCI in dogs, is not understood in detail so far; however, a fulminant and exaggerating secondary injury response with massive reactive oxygen species formation seems to be involved in this unique neuropathological entity. There are substantial gaps in the knowledge of pathological changes in IVDE with respect to more advanced and chronic lesions and the potential involvement of demyelination. Moreover, the role of microglia/macrophage polarization in IVDE-related SCI still remains to be investigated. A close collaboration of clinical neurologists and veterinary pathologists will help to facilitate an integrative approach to a more detailed understanding of the molecular pathogenesis of canine IVDE and thus to identify therapeutic targets.

## Introduction

Spinal cord injury (SCI) in dogs can be caused by either extrinsic or intrinsic forces. Though extrinsic traumatic forces such as road accidents, which make up the majority of human cases of severe SCI, do also occur in pet dogs, intervertebral disc extrusion (IVDE) is by far the most common cause for SCI in dogs (1). IVDE-induced SCI accounts for up to 2% of all diseases in dogs (2–4) and represents one of the most common diagnoses made by veterinary neurologists. In a study conducted in Switzerland with a referral hospital population of nearly 3,500 dogs with central nervous system (CNS) diseases included, IVDE represented the most common diagnosis, followed by epilepsy and other neurodegenerative diseases (5).

Due to high standards in clinical management, the prognosis of IVDE-induced SCI of mild to moderate severity is generally good; however, 40–50% of dogs with severe SCI secondary to IVDE (those who are paraplegic with absent nociception) do not recover the ability to ambulate and may be euthanized because of the condition, even with the highest standard of care. The post-mortem examination of such cases provides an opportunity to gain basic insights into the pathology and pathogenetic basis of this clinically important disease. Besides its doubtless high veterinary relevance, IVDE-induced SCI in dogs moreover shares striking similarities with human traumatic SCI (6). Similar to most cases of traumatic SCI in humans, IVDE-induced SCI is caused by a relative contribution of both compressive and contusive forces caused by structures anatomically located ventral to the spinal cord (6–8). This is in contrast to most experimental rodent models for SCI, which commonly rely on purely concussive injuries caused by dorsal weight drop or complete cord transection (6–9). Moreover, compared to rodents, the canine spinal cord more closely resembles the size of the human counterpart (8). Lastly, and probably most important, canine IVDE is a spontaneous disease with a high inter-individual variability (6, 7). Based on these similarities, canine IVDE has developed into an acknowledged translational animal model that may add the missing heterogeneity to experimental investigations in rodent models of SCI (6, 8). Consequently, veterinary clinical studies in canine SCI may help to translate findings from experimental rodent studies into the clinically relevant, naturally occurring disease (7, 8, 10–12).

An enormous body of literature exists on the morphologic and molecular pathology of experimental SCI, with comparatively less data on naturally occurring cases of human SCI. It is highly likely that canine IVDE-induced SCI shares many of these pathological features reported in both human traumatic SCI and experimental animal models. The present summary focuses on data that have been gained in (histo-) pathological studies on naturally occurring IVDE-induced SCI in dogs, referring to only a few studies on experimental SCI, where the knowledge of IVDE-induced canine SCI is only fragmentary or absent. Starting with a brief overview on canine intervertebral disc disease, the major focus of this paper is to provide an overview of the pathologic events in the injured canine spinal cord with reference to therapeutic implications where applicable.

### Basic Pathological Mechanisms of Canine Intervertebral Disc Degeneration

Degeneration of the intervertebral disc is commonly the prerequisite for later IVDE-induced SCI; i.e., IVD degeneration represents an important predisposing factor for the disc herniation into the vertebral canal. Early pioneer studies by Hansen (1952) (13) have extensively described the pathological changes during IVD degeneration and details of intervertebral disc anatomy and degeneration are reviewed elsewhere in this issue (Fenn et al.). Briefly, in chondrodystrophic dog breeds such as the dachshund, beagle, and Pekingese, the nucleus pulposus of multiple intervertebral discs undergoes progressive chondroid metaplasia beginning in juvenile individuals (2, 6). Initial degenerative changes are completed as early as 1 year of age (2, 4). Due to their familial predisposition, chondrodystrophic breeds are much more likely to develop disc herniation than non-chondrodystrophic breeds, as they are prone to premature senescence of the nucleus pulposus (6, 14). Among chondrodystrophic breeeds, French Bulldogs have gained enormously in popularity. Recent studies suggest that French bulldogs are prone to various neurological diseases with IVDE ranging on top of the neurological diseases in this breed (15). In contrast to other breeds, cervical location of IVDE seems to be more common in French bulldogs (15). The reasons for the relatively high level of predisposition for neurological diseases in this breed remain speculative; however, besides chondrodystrophy, excessive inbreeding might represent one factor that contributes to predisposition of French Bulldogs to neurological disease development (15). For a more detailed review of the genetic factors involved in canine IVDD the reader is referred to Dickinson et al. in this edition. Recent independent genome-wide association analyses for skeletal dysplasia and IVDE identified a highly expressed *FGF4* retrogene on CFA12, which is associated with both IVDE and chondrodystrophy (16). The nucleus pulposus is replaced by hyaline cartilage. The latter progressively degenerates and calcifies in the late stage of IVD degeneration (2, 17). In dachshunds with acute disc herniation, histopathology reveals that the majority of extruded disc material is calcified, even in the absence of radiographically visible calcification (18). More recent histopathological studies propose a grading scheme for intervertebral disc degeneration based on an assortment of parameters. These include morphology of the annulus fibrosus, chondrocyte metaplasia of the annulus fibrosus, tears and cleft formations, chondrocyte proliferation within the nucleus pulposus, presence of notochordal cells in the nucleus pulposus, matrix staining of nucleus pulposus with Alcian Blue/Picrosirius Red, endplate morphology, new bone formation, and subchondral bone sclerosis (19). In this study, glycosaminoglycan content and total histological score showed high correlation.

In contrast to chondrodystrophic breeds, the intervertebral disc of non-chondrodystrophic dogs has historically been believed to undergo an age-dependent and slowly progressing fibroblastic metaplasia of both the annulus fibrosus and nucleus pulposus (2, 4, 6), which may represent a non-hereditary wear-and-tear-phenomenon. This traditional concept, i.e., chondroid metaplasia of the nucleus pulposus in chondrodystrophic breeds, and fibrous metaplasia in non-chondrodystrophic dogs, has been recently been disputed by studies suggesting that IVDD in chondrodystrophic and non-chondrodystrophic breeds is more similar than previously believed (20). In fact, chondroid metaplasia is observed in both chondrodystrophic and non-chondrodystrophic dogs and fibrocytes were not seen in the nucleus pulposus in any of the investigated discs in a recent study, thus challenging this original “chondrodystrophic” and “non-chondrodystrophic” paradigm in canine IVDD (20).

### Clinical Spinal Cord Injury Caused by Intervertebral Disc Herniation

The clinical presentation of IVDE in dogs spans a spectrum ranging from neck or back pain to severe spinal cord injury with loss of sensory and motor function caudal to the lesion. While several clinical grading systems have been employed throughout the literature to quantify severity of injury, the most commonly used is a version of the modified Frankel scale. This scale ranges from paraplegia with absent superficial and deep pain sensation to normal dogs. Injury severity, as measured in this way, correlates well with prognosis for recovery after surgical decompression where dogs with grade 0 injuries experience return of unassisted ambulation and fecal and urinary continence in 50–60% of cases (21, 22).

The pathogenesis of canine IVD degeneration and IVDE has been recently reviewed in detail (14) and is also covered in detail in other articles in this issue. Using Hansen's descriptors, chondrodystrophic breeds are predisposed to Hansen type I herniation (IVDE) whereas the non-chondrodystrophic breeds are more prone to type II herniation (2, 17, 23). The vast majority of research focusses on IVDE, as it is the most common type and often induces the most severe lesions. Rapid extrusion of nucleus pulposus results in compressive and contusive injury to the spinal cord. Though IVDE induces a mixed contusive/compressive force to the respective spinal cord segment (6–8, 14), the extent of each varies both within the individual patient and with the type of herniation observed. Since Hansen type I disc extrusions typically occur acutely and with substantial force, they generally cause considerably more severe trauma to the respective spinal cord segments as compared to Hansen type II disc protrusions, which are less severe and lead to more slowly developing forces applied to the spinal cord (i.e., focus on the compressive part of the force) (2, 6, 13, 14, 23).

In chondrodystrophic breeds, approximately 75% of intervertebral disc herniations are found at the level of the Th 12 to L2 (13). Intervertebral disc herniations at the cervical level are less common, accounting for approximately 14 to 35 percent of all intervertebral disc herniations (4, 6, 23).

Some cases can clinically not be classified into either Hansen type I or type II, as a proportion of non-chondrodystrophic dogs may develop acute clinical signs with rapid onset, while few dogs with Hansen type I herniation (extrusion) may develop slowly progressing signs (14). Other types of herniation have been described and besides the aforementioned forms, in which IVDE is the sequela of IVD degeneration, non-degenerate physiological disc material may be herniated into the vertebral canal and/or spinal cord by extrinsic traumatic forces (traumatic disc prolapse) (13, 14, 24). Various other forms of IVD disease are covered in detail in the article of Fenn et al. in this Issue.

Irrespective of the exact type, herniation of the intervertebral disc typically occurs in the dorsal direction, i.e., into the vertebral canal (14). Monocytes and macrophages are found in extruded disc material, and there is activation of extracellular signal-regulated kinase p38 (25). Moreover, similar to IVDE in humans, canine thoracolumbar IVDE is associated with elevated gene and protein expression of key cytokines such as IL-6 and TNF-α and down-regulated expression of IL-1β (25).

## Pathology of Canine Intervertebral Disc Herniation-Induced Spinal Cord Injury

### General Morphology

Most of the data on pathological lesions in the spinal cord derive from individuals with acute to subacute severe SCI (paraplegic with and without pain perception, respectively), with a considerable lack of histopathological descriptions on more chronic lesions and less severe injuries, which is due to the fact that, in a non-experimental set-up of studies on a naturally occurring spontaneous disease, material for histopathological investigations most commonly derives from euthanized individuals with an acute onset of severe clinical signs and a poor prognosis. Thus, pathological descriptions are somewhat biased, and one should consider them mostly mirroring extreme cases of a wide spectrum of time course and lesion severity, respectively.

IVDE causes considerable, though highly variable, pathological alterations within the respective spinal cord segments and at distant sites within the neuraxis. Upon necropsy, dorsal removal of vertebral laminae exposes the vertebral canal and degenerate intervertebral disc material may be detected within the vertebral canal in close proximity and often firmly attached to the contused and compressed spinal cord segment. Macroscopic alterations in the spinal cord itself may range from no detectable changes, to discoloration, grossly obvious hemorrhage, severe spinal cord and dural laceration, or spinal cord atrophy in long standing cases. The histological changes observed in dogs with IVDE-associated SCI are relatively similar to histopathological alterations in spinal cords of humans affected by SCI, underlining the role of canine IVDE as a translational animal model that may allow extrapolation of findings to naturally occurring human cases.

**Figure 1:**
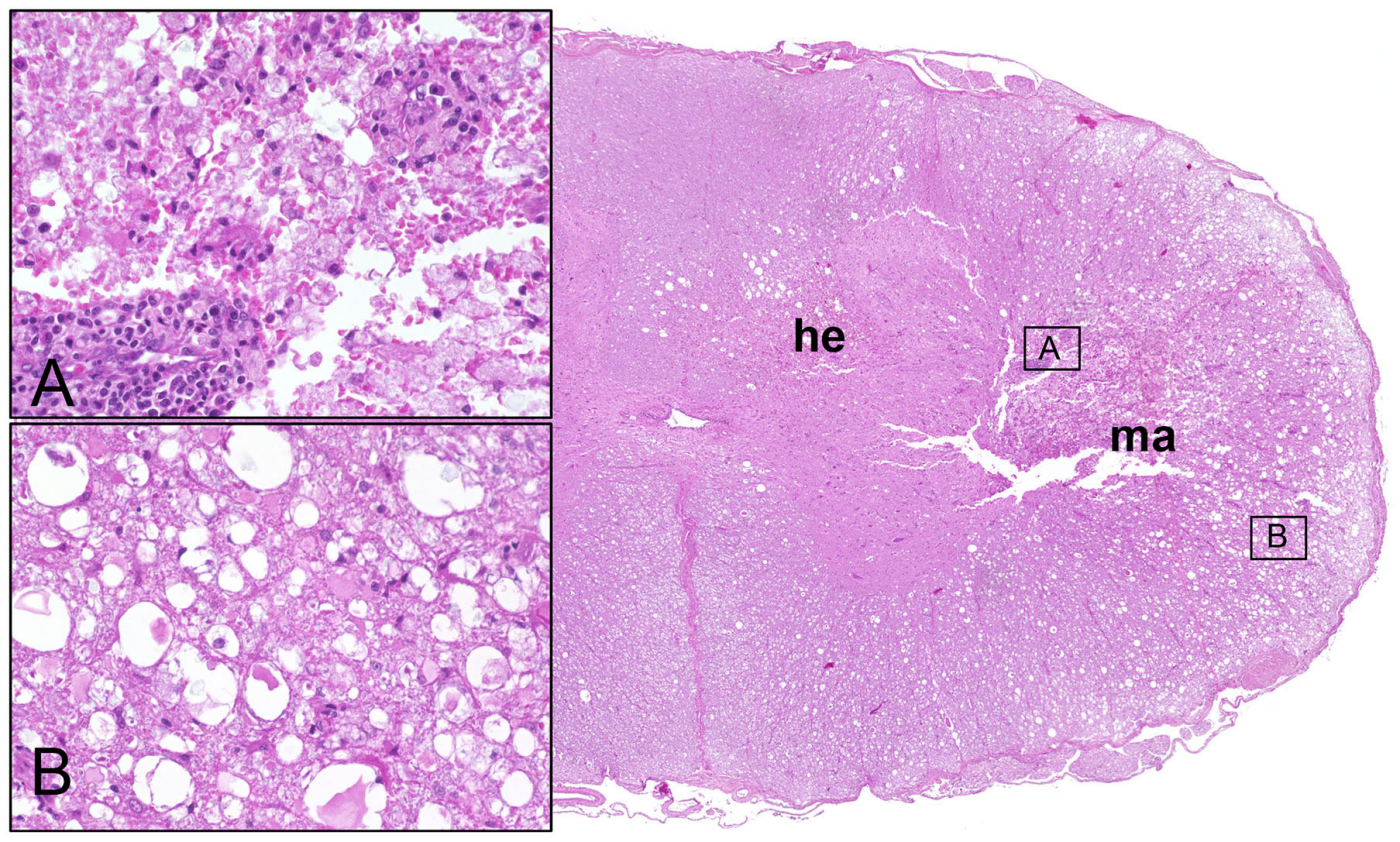
Male Dachshund with type I intervertebral disc herniation (acute extrusion). Overview (right side) of HE stained spinal cord transversal section with hemorrhage (he) accentuated within the gray matter and white matter malacia (ma). Inset upper left **(A)**: moderate perivascular cuffing of mononuclear leukocytes and focal disintegration of neuroparenchyma (necrosis, malacia). Inset lower left **(B)**: moderate to severe white matter vacuolation within the ventrolateral funiculus, characterized by multiple dilated myelin sheaths that contain hypereosinophilic swollen axons (spheroids). 20x magnification in insets.

Histopathological alterations in the spinal cord of dogs with SCI have been detailed as early as 1978 (26). In general, lesions are highly variable, and may consist of variable degrees of necrosis and hemorrhage in acute stages (26); Figure 1. Ultrastructurally, hemorrhages, axonal spheroid formation, glial cell swelling, white matter edema, and demyelination are observed in cases of naturally occurring canine SCI (9). Moreover, remyelination in the advanced disease by both oligodendrocytes and Schwann cells was shown using electron microscopy (9). Depending on the severity of the initial trauma, secondary injury processes may finally culminate into liquefactive necrosis (malacia) of the spinal cord segment and glial scarring with variable involvement of neuroparenchymal cavitation and cyst formation. Chronic intramedullary lesions/cavitations are associated with severe initial SCI and negative clinical outcome (27). Ascending and descending myelomalacia is a devastating complication in a proportion of dogs with SCI and will be discussed at the end of this chapter.

Clinical neurological grades of dogs affected by thoracolumbar IVDE-induced SCI correlate with the extent of white matter damage (28). Of interest, however, is the notable observation that clinical injury severity does not always correlate with severity of histopathologic lesions, underscoring the need for further studies of pathological features of canine IVDE-induced SCI (28). Additionally, some clinical signs such as duration of clinical signs, Schiff-Sherrington posture, loss of reflexes and pain on spinal palpation are not associated with the histopathological severity of spinal cord damage (28). These results suggest that some clinical signs are rather associated with functional neurological disturbances such as conduction block due to energy depletion or failure, that are not necessarily reflected by histopathological alterations.

Thus, both the immune response and axonal pathology are pivotal hallmarks of SCI (6). Consequently, these pathogenetic factors have been proposed to serve as major targets for future therapies (6, 29–32) and a detailed understanding of the underlying pathology during canine IVDE-induced SCI is a basis essential to the development of such therapeutic interventions (6).

### Axonal and Myelin Pathology in Canine IVDE-Induced SCI

Axonal damage is a central hallmark of all forms of endogenous or exogenous traumatic CNS injury and various studies have characterized the underlying molecular pathogenesis of axonal degeneration and regeneration in traumatic brain and spinal cord injury in detail (33). As axonal damage may be the most obvious pathological correlate of clinical motor deficits, it is not surprising that axonal damage is a consistent histopathological feature of canine IVDE-induced SCI. In histopathology, axonal damage generally appears as axonal swelling and the occurrence of hypereosinophilic spherical enlarged axons (spheroids, Figure 1) within dilated myelin sheaths. Sharing many pathogenetic features with Wallerian degeneration, axonal damage is not restricted to the lesion center at the site of disc herniation but may also be seen in various spinal cord segments cranial and caudal to the initial lesion site.

Ultrastructurally, axoplasmic changes in spinal cords from dogs suffering from IVDE-induced SCI are relatively similar to the ultrastructural axonal changes seen in experimental SCI in rodents and monkeys (9, 34–39). Following compressive injury to the spinal cord in rats there is periaxonal space formation, myelin disruption and granular disintegration of neurofilaments (35). Moreover, organelle accumulation and giant axons may occur (35). Contusion SCI in rhesus monkeys similarly leads to axonal accumulation of dense bodies, vesicular structures, multivesicular bodies, and organelles (36). Axoplasmic vesicles, mitochondria, and electron-dense bodies are observed within reactive axonal enlargements (37). Though variable, all of the above mentioned features are also observed ultrastructurally in dogs with IVDE-induced SCI (6, 9, 34).

Deficits in both fast anterograde axonal transport mechanisms and axonal neurofilament phosphorylation have been implicated in the pathogenesis of axonal damage in canine IVDE-induced SCI (6, 34). Using immunohistochemistry, β-amyloid precursor protein (APP) is not detectable in healthy axons due to fast axonal transport under physiological circumstances. However, there is fast accumulation of APP, if axonal transport is disturbed due to pathological conditions (40). Consequently, APP is a well-established immunohistological marker for axonal damage and has been previously used to detect damaged axons in experimental SCI in dogs caused by inflated angioplasty balloons, where its expression correlates with severity and duration of compression (41, 42). Similarly, experimental SCI in rodents and spontaneous SCI in people are both associated with strong axonal expression of APP (43–45).

**Figure 2:**
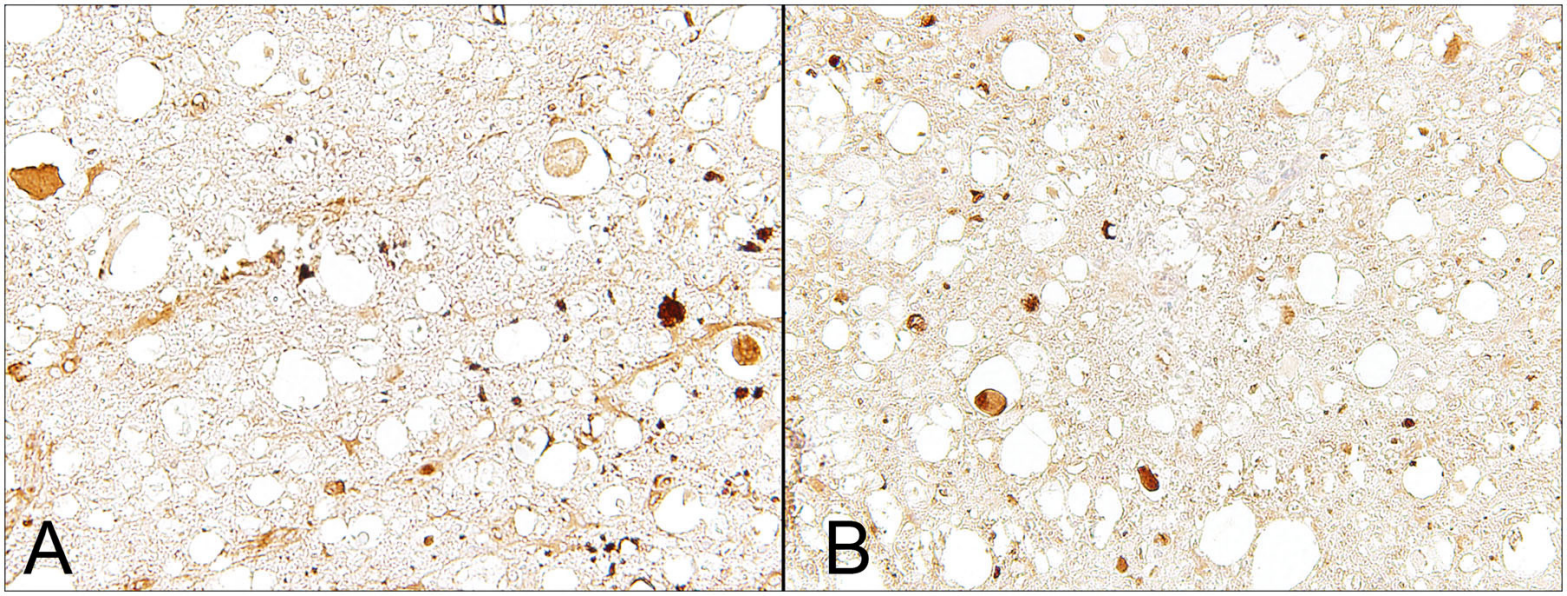
Male Dachshund with type I intervertebral disc herniation (acute extrusion). Immunohistochemical detection of axonal damage. **(A)** Beta-APP accumulates within swollen axons indicating disruption of the fast axonal transport machinery. **(B)** Non-phosphorylated neurofilament (nNF), another marker for axonal damage, is detected within numerous swollen axons but is also expressed by axons with a normal appearing diameter. 40x magnification.

In dogs with naturally occurring IVDE-induced SCI, APP is detectable in the lesion epicenter of both acutely and subacutely injured dogs (Figure 2) (6, 34). However, axonal APP expression can also be observed up to 3 cm caudal to the lesion epicenter during the subacute phase of injury, suggesting progressive spatial spread of disturbances in fast axonal transport (6, 34). Similarly, experimental rodent models and naturally occurring SCI in people leads to axonal APP-expression distant to the lesion epicenter (43, 45). These findings indicate that axonopathy is not simply and solely attributed to the initial primary injury but rather a timely and spatially progressive phenomenon reflecting secondary injury mechanisms (6).

In parallel to axonal APP-immunoreactivity, previous immunohistochemical studies on canine IVDE-induced SCI revealed enhanced axonal expression of non-phosphorylated neurofilaments (n-NF) in axons of dogs with acute and subacute SCI (6, 34); Figure 2. Moreover, similar to APP, n-NF-expression has been noted in axons several centimeters apart from the lesion center (34). However, in contrast to APP, which is mainly detected in swollen axons, n-NF immunopositivity was also seen in several axons with normal diameters (Figure 2) (6, 34). This implies that both markers might label, at least in part, distinct axonal pathological processes (6). In traumatic brain injury in rats, neurofilament compaction in axons has previously been reported to occur independently from APP-immunoreactivity (46). Thus, neurofilament alterations and disturbed axoplasmic transport might in part represent differing pathological phenomena (6).

In addition to traumatic CNS injury, enhanced axonal n-NF and APP-expression has been demonstrated in several animal models of demyelinating disease in various species including some dog studies (47–50), suggesting that altered neurofilament phosphorylation and disturbances in fast axonal transport represent conserved phenomena of axonopathy irrespective of the underlying disease entity.

Though axonal damage predominates, evidence for intrinsic axonal regeneration attempts has been reported in dogs with IVDE-induced SCI in terms of axonal expression of growth-associated protein 43 (GAP-43) (34). GAP-43 immunoreactivity was noted in a small proportion of axons in dogs with acute and subacute SCI, which vas verified by immune-electron microscopy. Ultrastructurally, immunoreaction was noted in swollen axons lacking dense body accumulation, but filled with large numbers of mitochondria (34). Axons express GAP-43 during development and regeneration (51). Live imaging on individual axons in experimental SCI have shown early axonal regeneration attempts (52); however, functional restoration seems to be insufficient. It is proposed that regenerating axons during SCI may fail to navigate to a proper target (52). This might in part be attributed to the expression of regeneration-inhibiting molecules such as Nogo and MAG, and pharmacological modulation of these molecules is believed to represent a promising target to facilitate axonal regeneration in terms of functional restoration (53, 54). Several further experimental therapeutic approaches aim to facilitate these intrinsic regenerative responses (29). In fact, transplanting regeneration promoting cells into the spinal cord of rodents with SCI has shown to enhance axonal GAP-43 immunoreactivity, which was associated with an improved clinical outcome (55–57). Moreover, facilitation of alternatively activated anti-inflammatory macrophages is paralleled by increased axonal expression of GAP-43 and improved locomotor recovery in spinal cord lesioned mice (58).

Myelin pathology, though a focus of experimental SCI work, has not been extensively reported in canine SCI. Though there is reduced immunoreactivity of myelin basic protein (MBP) in the white matter of dogs with subacute IVDE-induced SCI (34), this has rather been attributed to myelin edema and myelin sheath swelling than true demyelination. In an ultrastructural study of canine SCI, including various causes such as fractures, subluxations, and IVDE, demyelinated axons were observed within 2 weeks after initial injury and, interestingly, in advanced disease stages, both Schwann cell and oligodendrocyte remyelination was observed (9). Moreover, subtle partial and paranodal myelin abnormalities were seen ultrastructurally. This evidence for delayed myelin loss in canine IVDE-induced SCI is mirrors the situation in human spinal cord injury. Naturally occurring SCI in humans is associated with delayed and long-lasting myelin loss (59, 60). Morphologically detectable myelin abnormalities are generally observed subsequent to early axonal damage, thus recapitulating the principle processes during Wallerian degeneration. Moreover, demyelination in canine SCI might in part also reflect pathomechanisms referred to as the “inside-out theory” in neurodegenerative diseases (6, 61). Though this concept is controversial, it suggests that axonal damage functions as a mechanism triggering secondary demyelination (47, 61). Several lines of evidence indicate similarities in terms of this triggering function of primary axonopathy between neurodegenerative and viral CNS diseases on the one side and SCI on the other side (6, 61). In a clinical context, dogs with thoracolumbar IVDE with loss of ambulation had higher MBP concentration within the CSF compared with control dogs, suggesting that elevated MBP levels within the CSF are associated with poor clinical outcome (62).

Based on the assumption that demyelination is an event that occurs relatively late in the progress of secondary injury, investigations on chronic cases of canine IVDE-induced SCI are urgently needed. Evidence that demyelination does occur in chronically injured dogs is for instance based on clinical trials. 4-Aminopyridine (4-AP) is a compound known to improve function in demyelinating conditions. Dogs with spinal cord injury treated with 4-AP show significant improvement in supported stepping scores (63) suggesting that demyelination plays a role in advanced and long standing cases. As mentioned above, pathological data on naturally occurring canine SCI are primarily based on dogs with acute to subacute IVDE-induced SCI but there is little information upon the histopathology of chronic cases (14, 64). Similar to experimental data and lesion pathology in human SCI, chronic cases of canine IVDE-induced SCI are characterized by progressive white and gray matter loss with or without cyst formation and progressive replacement by extensive gliosis (14). In an MRI-study on chronic SCI in dogs, intramedullary cavitation and cyst formation was reported (27). Histopathologically, chronic lesions were characterized by gray matter-accentuated malacia, severe gliosis, and variable infiltration of phagocytic gitter cells (27). Multiple axonal spheroids can be detected, suggesting ongoing axonal damage. Myelin sheaths within the white matter showed dilatation and occasional myelinophages within dilated myelin sheaths. Some cases exhibited pan-myelomalacia with complete loss of organotypic structure, replaced by diffuse extensive gliosis (27). Mirroring overall neuroparenchymal loss of both gray and white matter, macroscopic changes of the chronically injured canine spinal cord may include hour-glass shaped atrophy of the respective spinal cord segment (27). Similarly, in experimentally induced SCI in dogs histological analyses at 12 weeks after SCI revealed amorphous cavities in the gray matter with spread to the white matter with caudally accentuated spatial spread up to 1 cm apart from the epicenter (65).

### Inflammation and Glial Cell Reactions in Canine IVDE-Induced SCI

**Figure 3:**
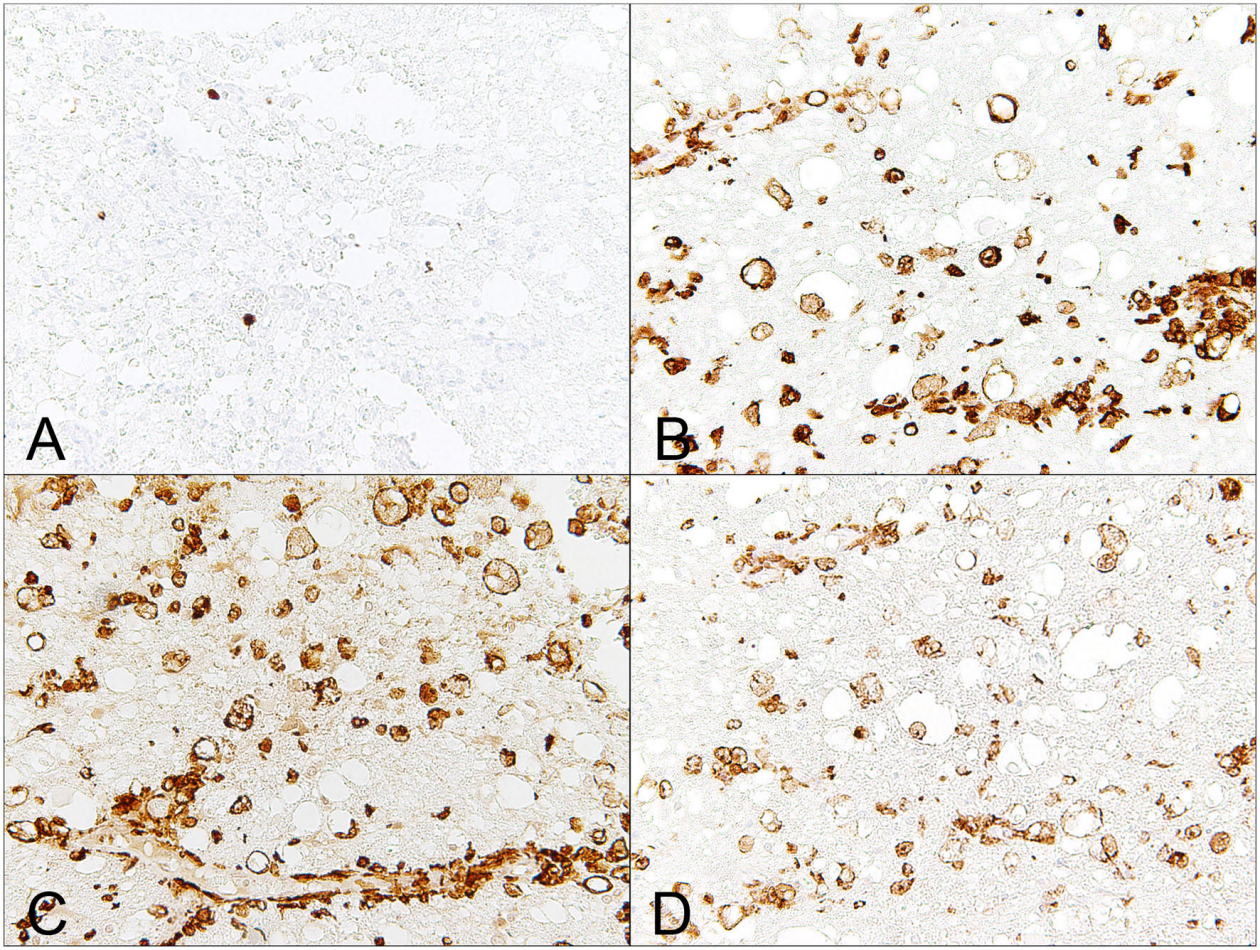
Male Dachshund with type I intervertebral disc herniation (acute extrusion). Immunohistochemical detection of macrophages, which are a dominating immune cell population involved in secondary injury mechanisms. **(A)** Mac387, a clone that detects myleoid/histiocyte antigen, only detects relatively few, monocyte-like blood born macrophages. **(B)** There is severe up-regulation of MHC class II on phagocytic gitter cells. **(C)** Similarly, Iba-1, a pan-macrophage marker, labels numerous phagocytic microglia/macrophages within the affected white matter. **(D)** CD204, a marker that has been proposed to mainly detect M2-polarized macrophages, labels several microglia/macrophages within the white matter and within dilated, optically empty myelin sheaths (myelinophagia). 40x magnification.

In severe acute cases of canine SCI, the first cell type that arises are neutrophils, and increased cell numbers of neutrophils are commonly detected within the CSF of dogs with IVDE-induced SCI. Histopathologically, neutrophils are commonly associated with areas of hemorrhage (66). In parallel, there is infiltration of MAC-387-positive monocyte-derived macrophages and variable perivascular leukocyte cuffing (Figures 1, 3). Cellular reactive changes begin to be more obvious in subacute cases, in which there is a phagocytic response that is impressively dominated by microglia/macrophages (66). MHC class II expressing microglia/macrophages have also been reported as the predominating cell type in human SCI (Figure 3) (67), whereas lymphocytes seem to play a subordinate role (67, 68). In dogs, microglial cells have been analyzed in detail in various neurological diseases such as canine distemper virus infection and SCI (11, 69, 70). In healthy dogs, canine microglia derived from the spinal cord show a relatively higher capacity of phagocytosis and generation of reactive oxygen species (ROS) as compared to cells derived from the healthy brain (70). Dogs with SCI reveal enhanced microglial expression of surface molecules such as B7-1, B7-2, MHC class II, CD1c, ICAM 1, CD14, CD44, and CD45, as determined by flow cytometry (11). Besides, phagocytosis and ROS generation of microglia are elevated in dogs with SCI (11).

Extensive research on microglia/macrophages is similarly done in experimental laboratory studies of SCI and manipulation of the response of these cells is regarded as a promising field in the development of new therapeutic approaches. Based on a relatively novel basic, but very simplified concept that microglia/macrophages may be polarized into either pro-inflammatory and neurotoxic (M1-) cells or alternatively activated, anti-inflammatory and regeneration promoting (M2-) cells, a bulk of experimental research has been conducted focusing on the role of these cells in SCI. Pioneer studies on rodent SCI revealed that SCI is characterized by an early and persisting M1-dominated macrophage response (71). The fact that this polarized M1-response overwhelms a relatively sparse M2-macrophage response has led to the idea that shifting this phenomenon toward a regeneration-promoting M2-dominated response might be a rewarding research target for therapies in SCI (71).

Whether this macrophage polarization also occurs in the context of clinically relevant naturally occurring canine SCI has not been investigated to date. However, several lines of evidence indicate that the microglia/macrophage response is similarly associated with a polarization of macrophages toward a pro-inflammatory phenotype. Subacute canine IVDE-induced SCI is associated with a dominating response of MHC class II-expressing phagocytic microglia/macrophages that is paralleled by a pro-inflammatory microenvironment (66, 72). Moreover, microglia/macrophages are a pivotal source of ROS, tissue degrading metalloproteinases and neurotoxic mediators.

Detection of M1- and M2-macrophages *in situ* relies on immunohistochemical markers and there is a well-established panel of such antibodies for the distinctive detection of these cells in laboratory rodent tissue. However, the markers routinely used for the detection of rodent M1- and M2-macrophages cannot simply be transferred to other species. The nitric oxide and arginase metabolism of macrophages is a commonly used basis for the detection of rodent M1- and M2 macrophages. Consequently, arginase (Arg)1 and inducible nitric oxide synthase (iNOS) are the prototype markers to detect rodent M2 and M1-macrophages in tissue sections, respectively (71). However, there are considerable species differences, especially in the context of NO metabolism of macrophages and these well-established markers are not necessarily adequate to detect human and canine macrophages (73). Thus, development of a panel of antibodies that enables the detection of canine M1- and M2-macrophages in tissue sections is highly needed. Recently, canine polarized macrophages have been characterized *in vitro*. Unstimulated (M0), M1- (GM-CSF, LPS, IFNγ-stimulated) and M2- (M-CSF, IL-4-stimulated)-polarized canine blood-derived macrophages showed distinct ultrastructural morphologies (73, 74). Interestingly, immunofluorescence using standard literature-based prototype-antibodies against CD16, CD32, iNOS, MHC class II for the detection of M1-macrophages and CD163, CD206, and arginase-1 for the detection of M2-macrophages demonstrated that solely CD206 was an appropriate marker that discriminated M2-macrophages from both other phenotypes (73, 74). In the same study, a global microarray analysis was performed and revealed changes in the transcriptome of polarized canine macrophages and similar to the results on the protein level, there were only minor overlaps in the gene sets of the dog compared to prototype markers of murine and human macrophages (73, 74). The transcriptome data of these canine macrophages might represent a basis for the subsequent development of immunohistochemical markers for the distinction between canine M1- and M2-macrophages, respectively, that are highly needed to classify the microglia/macrophage phenotype in the naturally injured canine spinal cord.

As mentioned above, data on naturally occurring chronic IVDE-induced lesions are extremely sparse. Glial scar formation is a common finding in experimental and naturally occurring human SCI. Similarly, extensive glial proliferation (gliosis) has been reported in dogs with chronic IVDE-induced SCI (27). Experimental SCI in dogs 12 weeks post injury is similarly characterized by severe astrogliosis as revealed by enhanced immunoreactivity for GFAP with spatial spread, mainly in the caudal direction (65).

Ependymal cells have recently been highlighted to participate in the cellular reaction following canine SCI. Due to its function as a source for neural precursors the spinal ependymal layer is believed to possess regenerative capacity and consequently represents another field of growing research, especially in the context of SCI (75, 76). Immunohistochemistry revealed increased numbers of GFAP-positive cells in acute IVDE-induced SCI in dogs with SCI at the lesion epicenter and additionally at sites proximal to the lesion center (76). It is proposed that the spinal ependymal layer may have the capacity of astrocytic differentiation during naturally occurring SCI in dogs. Besides enhanced GFAP-immunoreactivity of the spinal ependymal layer, acute IVDE-induced SCI is also characterized by altered E-cadherin expression patterns, indicating that a loss of cellular polarity could promote ependymal cell migration to the injury site (76).

### The Need for Non-invasive Biomarkers in Canine IVDE Induced SCI

From a clinical perspective, a non-invasive biomarker that is able to predict clinical outcome, particularly in dogs with the most severe SCI, is highly needed. Multiple studies have assessed molecules in the CSF or serum, based on the hypothesis that the concentration of such metabolites is associated with injury severity and outcome, respectively. The results of these studies are also interesting from a pathological point of view, as clinically detected elevated levels of serum and CSF molecules may also be assessed in pathological analyses on post-mortem tissue such as immunohistochemistry and RT-qPCR methods. Vice versa, evidence from pathological studies may be extrapolated to clinical settings as enhanced expression of molecules detected via histopathological methods or molecular biology on post mortem tissue might develop new hypotheses in the search for novel biomarkers in a clinical setting. Thus, research on biomarkers for IVDE-induced SCI is an interesting field in which pathology and clinical neurology obviously benefit from each other.

Previously assessed candidate biomarkers in canine IVDE-induced SCI in CSF and serum, respectively, include metalloproteinases, neuronal/axonal cytoskeletal molecules, inflammatory cell counts, acute phase proteins, cytokines, arachidonic acid metabolites, and glial cytoskeletal components.

Enhanced MMP-9 activity in the CSF of the lumbar spine has been reported to indicate severe SCI with poor prognosis (77). Similarly, microtubule-associated protein tau, detected by ELISA in cisternal CSF, is associated with unsuccessful outcome in paraplegic dogs suffering from thoracolumbar or cervical IVDE (78). Serum levels of phosphorylated neurofilament heavy chain (pNF-H) are associated with severity of thoracolumbar IVDE and may predict an unfavorable prognosis (79). Increased cisternal CSF total nucleated cell count correlates with injury severity; however, the investigated CSF characteristics did not differentiate IVDE-induced SCI from other spinal cord diseases (80). The CSF concentrations of the acute phase proteins C-reactive protein and haptoglobin are associated with IVDE-induced injury severity; however, not correlating with 42 d motor outcome (81). The concentration of the arachidonic acid metabolites PLA2 and PGE2 in the CSF are higher in dogs with SCI compared to control dogs, while LCT4 concentration is lower in dogs with SCI than that in control dogs (82). Moreover, the concentration of PGE2 positively correlates with increased severity of SCI. Within the 1st days of IVDE-induced SCI, serum levels of GFAP and S100β rapidly rise, while pNF-H showed a later peak at 14 days post injury (83). Moreover, serum GFAP levels during the first 3 days can be used as a biomarker to predict recovery in severe SCI (83).

### Matrix Metalloproteinases in Canine IVDE-Induced SCI

Matrix metalloproteinases (MMPs) have been shown to participate in the pathogenesis of canine IVDE-induced SCI in several studies. MMPs play a pleiotropic role in various neurologic diseases. They are involved in both axonal degeneration and regrowth and their signaling is crucial for postinjury reorganization and synaptic stabilization (84). Besides, MMPs are pivotal mediators of secondary injury and promote disruption of the blood-brain and blood-spinal cord barrier (84). In parallel, their signaling is necessary for healing processes such as angiogenesis, but on the other hand MMP expression promotes formation of a regeneration-inhibitory glial scar. Thus, MMPs play an important pathogenetic role during SCI.

Especially, the gelatinases MMP-2 and MMP-9 show time-dependent expression during SCI in both experimental and naturally occurring SCI (68). MMP-9 knock-out mice show less expression of regeneration inhibiting molecules when compared to wild-type mice with SCI (85). MMP-9 has thus gained much attention as a therapy target, as modulation of its expression might reduce glial scarring following SCI (85). In acute human SCI, MMP-9 is expressed by neutrophils in areas with hemorrhage as revealed by immunohistochemistry (68). Rapidly enhanced expression of MMP-9 in experimental contusion SCI in rodents is associated with an inappropriate function of the blood-spinal cord barrier as well as in inflammation and locomotor recovery (86). Compared to wild type mice, there is improved locomotor recovery in MMP-9 knock-out mice (86, 87).

Following experimental SCI in rodents, there is also upregulation of MMP-2. However, this upregulation is delayed when compared to MMP-9 (88, 89). In contrast to MMP-9, deficiency in the expression of MMP-2 is associated with impairment of locomotion in experimental SCI in mice (88). Thus, it is proposed that MMP-2 rather plays a beneficial role following SCI, in part by at regulating function that seems to target axonal plasticity and white matter sparing (88).

Dysregulation of the gelatinases MMP-2 and MMP-9 has been reported in previous studies on canine IVDE-induced SCI by means of RT-qPCR on spinal cord tissue of dogs (6, 34). While MMP-9 transcripts were up-regulated in dogs with acute SCI, MMP-2 exhibited a transient downregulation in the acute disease phase as compared to spinal cord tissue of neurologically healthy dogs (6, 34). Similarly, MMP-9 activity is increased in the CSF and serum of dogs with acute IVDD as revealed by zymography (90). Interestingly, elevated MMP-9 levels are associated with a poor outcome in dogs with IVDE-induced SCI (77, 90). Based on these observations and the hypothesis of a detrimental role of early MMP-9 signaling in dogs with IVDE-induced SCI, a randomized, blinded, placebo-controlled study was initiated to assess efficacy of the broad spectrum MMP-inhibitor GM6001 (91). In this study, dogs received GM6001 dissolved in dimethyl sulfoxide (DMSO), DMSO alone, or saline. GM6001 reduced serum MMP-9 activity compared to the other two groups (91). Interestingly, dogs treated with saline had significantly lower functional scores than dogs receiving DMSO or GM6001, demonstrating that there was no independent effect of GM6001 (91). The authors conclude that DMSO might have therapeutic effects in the acutely injured spinal cord. Similarly, recent clinical trials using the same agent, GM6001, demonstrated higher bladder compliance in dogs treated with GM6001 and DMSO as compared to controls (92). However, there were transient greater adverse events in GM6001-treated dogs compared to those treated with the vehicle control, and again, there was no difference in motor scores between dogs treated with GM6001 and DMSO vs. dogs treated with DMSO alone (92).

### Cytokines in Canine IVDE-Induced SCI

The cerebrospinal fluid of dogs with acute, surgically treated, thoracolumbar IVDE has been assessed regarding expression of interleukin (IL)-2,−6,−7,−8,−10,−15,−18, granulocyte macrophage colony stimulating factor (GMCSF), interferon gamma (IFN-γ), keratinocyte chemoattractant-like (KC-like) protein, IFN-γ-inducible protein-10 (IP-10), monocyte chemotactic protein-1 (MCP-1), and tumor necrosis factor alpha (TNF-α) (93). Using a bioplex system, IL-8 concentration was found to be significantly higher in SCI cases than healthy controls and negatively correlated with the duration of SCI (93). Moreover, the MCP-1 concentration demonstrated to be negatively associated with 42-days post-injury outcome (93). Similarly, an early upregulation of pro-inflammatory cytokine mRNA (IL-6, IL-8 and TNF) has been noted in spinal cord tissue of dogs with acute IVDE-induced SCI (1–4 days post IVDE) using RT-qPCR of mRNA extracted from affected spinal cord tissue (66). IL-8 mRNA upregulation was also found in dogs with more than 4 days post IVDE suggesting a prolonged role of this pro-inflammatory cytokine in the pathogenesis of canine IVDE-induced SCI (66, 93). While IL-10 showed no differences in expression in either control dogs or dogs with SCI, expression of TGF-β showed up-regulation exclusively in spinal cord tissue of dogs with subacute SCI for more than 4 days. It is concluded that acute IVDE-induced SCI in dogs is dominated by a pro-inflammatory microenvironment (66, 72). The previous findings on cytokine expression in canine IVDE-induced SCI largely mirror findings in human cases of SCI and experimental SCI in rodents. For instance, several pro-inflammatory cytokines including IL-6 and IL-8 have also been reported to be upregulated in the CSF of humans affected by SCI (94). Interestingly, IL-8 levels within the CSF of people with SCI positively correlate with injury severity (94, 95). The delayed expression of TGF-β in dogs with IVDE-induced SCI is in concordance with experimental SCI in rats (96). TGF-β reduces the lesion volume and is associated with decreased numbers of macrophages in experimental rat SCI (96, 97)

Taken together, there is dysregulated cytokine expression with a lack or delay of anti-inflammatory cytokines and a dominance of pro-inflammatory cytokines during acute canine IVDE-induced SCI. These factors are thus believed to contribute to the lesion development and secondary injury processes in canine IVDE-induced SCI (6).

Further demonstrating that pro-inflammatory processes predominate in acute IVDE-induced SCI in dogs, there is significant dysregulation of acute phase proteins in the CSF of dogs with IVDE-induced SCI. Concentrations of C-reactive protein (CRP), haptoglobin (Hp), alpha-1-glycoprotein, and serum amyloid A were measured in a previous study (81). Interestingly, compared with healthy control dogs, Hp concentrations were higher in the CSF of affected dogs (81). Moreover, the authors reported that higher concentrations of CRP and Hp were associated the severity of injury; however, CSF APP concentrations and 42 d motor outcome did not reveal significant correlation (81).

## Ascending and Descending Myelomalacia

A small but significant proportion of dogs affected by IVDE may develop one of the most disastrous complications, progressive myelomalacia [PMM; (98)]. PMM is a unique entity, observed in both humans and dogs with severe injuries to the spinal cord and distinct from the initial SCI event. Though PMM can be observed following various forms of SCI including external trauma such as fractures, IVDE represents the most common initial type of SCI in dogs with subsequent PMM. The condition is characterized by progressive hemorrhagic necrosis of the spinal cord that diffusely ascends and/or descends over many spinal cord segments (99). PMM often develops early during the time course of IVDE and most dogs with PMM are euthanized within 3 days after onset of signs due to progressive respiratory paralysis (100). Considerable efforts have been undertaken to identify risk factors that are associated with this typically fatal condition. The prevalence of PMM is as low as 2% in the overall dog population with thoracolumbar IVDE, but severely elevated in paraplegic dogs that lack pain perception (101). In fact, the prevalence of PMM rises up to 10–12% in paraplegic dogs with absent deep nociception (21, 102). It appears that French Bulldogs may possess a breed predisposition to develop the devastating condition and the condition is more commonly diagnosed in dogs with extensive hyperintensity of the spinal cord on T2 weighed magnetic resonance imaging, dogs < 6 years of age, dogs with L5-6 disc herniations, and dogs with a rapidly progressive onset of clinical signs (103). A comprehensive recent study on 45 dogs with PMM identified IVDE at the lumbar intumescence as a strong risk factor that was associated with PMM (104). Moreover, surgery performed more than 12 h after loss of ambulation was also positively and treatment with corticosteroids was negatively associated with the development of PMM (104). Serum levels of GFAP have also been proposed as a biomarker for PMM. In one study, of which seven dogs had detectable levels of serum GFAP, 6 developed PMM (105). Sensitivity and specificity of the GFAP to predict PMM were reported to be 75 and 97.7%, respectively (105).

While there have been some advances in the identification of risk factors associated with the disease, knowledge on the pathogenesis of PMM is strikingly sparse. In a histologic study, endothelin-1 (ET-1) immunoreactivity was noted in astrocytes, macrophages, and neurons, but only rarely in endothelial cells (106). At the lesion epicenter of spinal cord hemorrhage, ET-1 immunoreactivity was significantly higher in astrocytes and lower in neurons than in non-affected control dogs. Moreover, there was higher astrocytic and neuronal ET-1 immunoreactivity in spinal cord segments remote from the epicenter than in the center itself. The authors conclude that elevated ET-1 expression over multiple spinal cord segments after IVDE might be involved in the pathogenesis of PMM (106).

**Figure 4:**
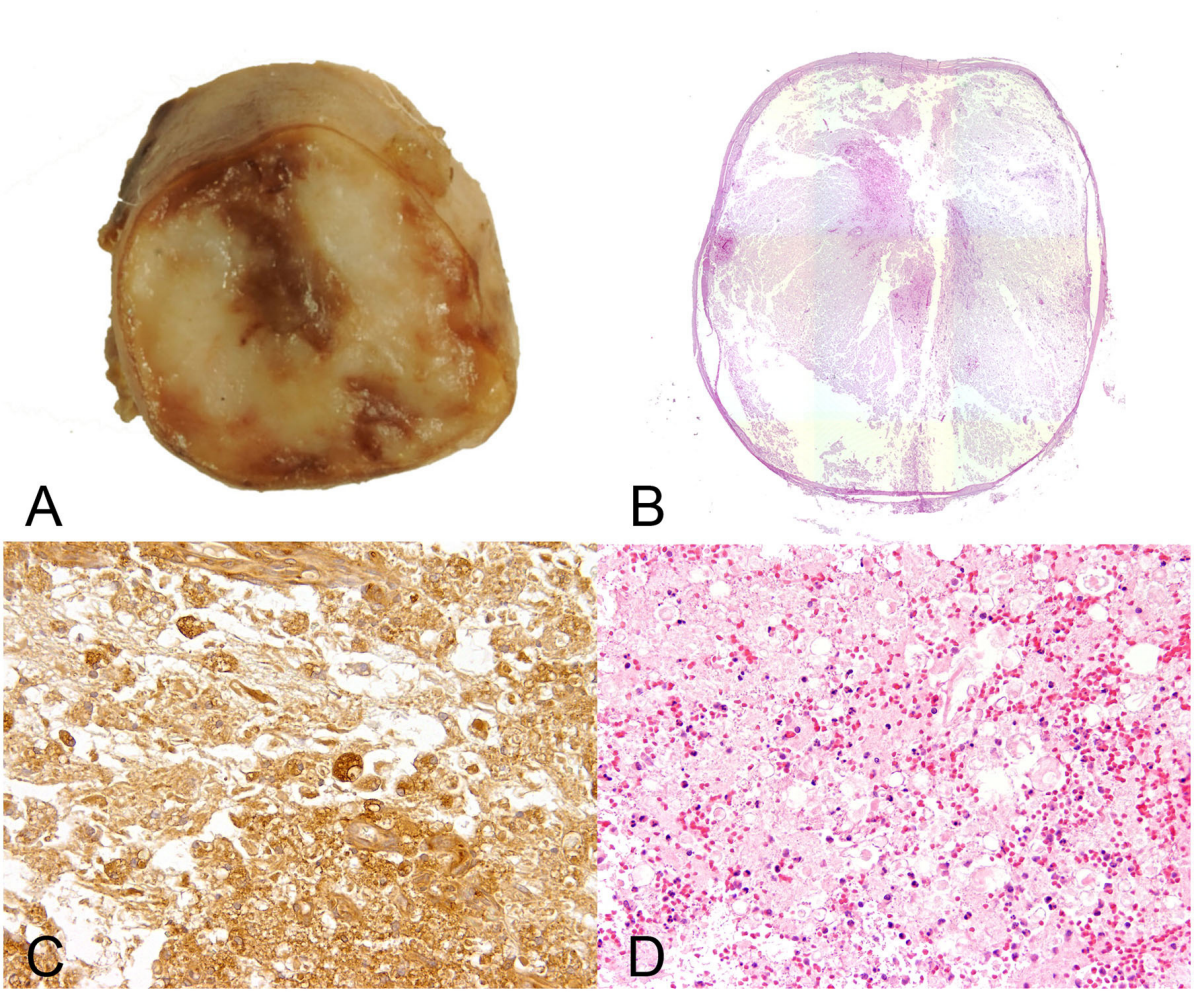
Male 6 years old Yorkshire Terrier with progressive myelomalacia (PMM) following acute intervertebral disc extrusion. In PMM the shown lesions are not restricted to the initial site of spinal cord injury but extend several centimeters into cranial and caudal direction (ascending and descending malacia). **(A)** Gross picture of a transversal section of the formalin fixed spinal cord with complete disintegration of spinal cord neuroparenchyma and hemorrhage. **(B)** The HE stained overview of the transversal section shows polio- and leukomyelomalacia with complete loss of cellular details and loss of distinction between white and gray matter. **(C)** Multiple foamy microglia/macrophages labeled by the lectin of Bandeiraea simplicifolia 1 have infiltrated the lesion and remove cellular debris. 40x magnification. **(D)** There is severe extravasation of erythrocytes within the white and gray matter (hemorrhage), associated with infiltration of viable and degenerate neutrophils adjacent to areas of white matter damage with spheroids and myelin vacuolation. 10x magnification.

Histopathologic alterations of PMM are generally characterized by severe liquefactive necrosis of the spinal cord that extends over several segments (Figure 4). It is proposed that PMM represents a form of exuberant and dysregulated secondary injury response (99). The affected spinal cord tissue shows extensive hemorrhage and necrosis in both the gray and white matter with disruption of myelin and necrotic and chromatolytic neurons as well as prominent swollen endothelial cells lining remaining blood vessels (Figure 4) (99). Parenchymal and meningeal blood vessels have been reported to be necrotic with perivascular deposition of fibrin (98). Moreover, some vessels may contain thrombi (98). Severe lesions are characterized by an amorphous mixture of tissue debris, macrophages, and blood (106). Variably, intervertebral disc material may be detected in proximity to the meninges. The necrotic processes are accompanied by a reactive inflammatory response with neutrophils predominating due to the acute nature of the pathologic alterations. Moreover, lesions are characterized by infiltration of CD18-positive phagocytic microglia/macrophages (99). Hemorrhagic and necrotic debris may also be detected within the central canal in spinal cord segments remote from the lesion epicenter (107). In fact, intramedullary and subdural hemorrhages are significantly associated with the degree of white and gray matter damage, and the progressive nature of PMM is in part thought to be linked to high intramedullary pressure (107).

Oxidative stress is proposed to be involved in the pathogenesis of PMM, evidenced by the fact that PMM is associated with elevated levels of 8-isoprostanes and acrolein with concurrent reduction in endogenous anti-oxidation of glutathione in the CSF and urine of dogs suffering from the disease (99). The authors propose that the pathological condition of PMM represents an extreme case of secondary injury, in which the physiological defense systems are unable to terminate the progression of oxidative injury (99). Moreover, decreased anti-oxidation is associated with increased phagocytosis at the lesion center (99), suggesting that macrophages that occur during PMM might play a detrimental role in the process. The role of macrophages, and especially their potential polarization toward a neurotoxic phenotype, has not been addressed in detail so far and might represent a promising field for future studies. However, infiltration of neutrophils and macrophages has so far been regarded as a bystander phenomenon, that is not initiating the progression of PMM by itself (99).

## Conclusions And Future Directions

Conclusively, in parallel to the ongoing and growing focus on IVDE as a translational clinical animal model for SCI, there is a growing number of publications, investigating pathologic processes that occur following herniation of the intervertebral disc into the vertebral canal. Morphologic, axonal, glial and immune responses largely mirror changes seen in other animal models for SCI and the human disease; however, despite its high prevalence in veterinary clinical neurology, relatively little is known on the exact time course of secondary injury processes in the canine spinal cord affected by SCI. As immune processes, and here, especially the role of microglia/macrophages, is a rapidly growing field of experimental SCI research and a promising target for novel therapeutic approaches, further focus on the role of this cell population in this clinically relevant SCI model appears highly interesting for future histopathological and molecular studies. This will involve the establishment of immunohistochemical markers that are distinctive in the detection of different canine macrophage polarization stages (i.e., M1 and M2 macrophages). Moreover, integration of clinical and pathologic data in order to get detailed insights into the time course of immune responses and axonopathy, will provide an opportunity to improve the prediction of outcome and identify potential therapy targets. The same is true for the search of biomarkers, where close integrative collaboration of basic pathology science and research in the clinical setting will profit from each other in order to identify predictive factors influencing the course and outcome of IVDE-induced SCI.

The considerable paucity of pathologic data on chronic and advanced disease stages demonstrates the necessity of pathological investigations of such cases. This involves routine sampling of spinal cord tissue during necropsy, also from cases without an acute neurologic disease history from the side of pathology and rigorous communication of anamnestic data, as a considerable number of dogs might undergo necropsy due to other acute diseases where spinal cord alterations subsequent to IVDE years ago may be overseen.

Lastly, the pathogenesis of PMM as a devastating complication of canine SCI, is incompletely understood. There are surprisingly few studies on the pathology of this exacerbated form of secondary injury. The ongoing development of a panel of immunohistochemical methods for the detection of secondary injury processes such as immune and glial response and axonal damage including molecular alterations in research of canine IVDE induced SCI provides a promising tool for investigations on PMM. This will help to identify commonalities and differences and potentially contribute to the identification of predictive biomarkers and more detailed understanding of the pathogenesis of PMM.

## Author Contributions

All authors listed have made a substantial, direct and intellectual contribution to the work, and approved it for publication.

## Conflict of Interest

The authors declare that the research was conducted in the absence of any commercial or financial relationships that could be construed as a potential conflict of interest.
